# Association between asthma and periodontitis

**DOI:** 10.6026/973206300200059

**Published:** 2024-01-31

**Authors:** Wael I Ibraheem, Ashok Kumar Bhati, Fai Mohammed Essa Ageeli, Rehab Abdu Sufyani, Maram Ahmed Darraj, Enas Omar Ageeli, Khalid Mohammed Mobarki, Majed Yahya Alhazmi, Saiid Elshafey Mohamed Beshir

**Affiliations:** 1 Department of Preventive Dental Sciences, College of Dentistry, Jazan University, Jazan 45142, Saudi Arabia; 2 College of Dentistry, Jazan University, Jazan 45142, Saudi Arabia; 3 Department of Maxillofacial Surgery and Diagnostic Sciences, Division of Oral and Maxillofacial Surgery, College of Dentistry, Jazan University, Jazan 45142, Saudi Arabia

**Keywords:** Periodontitis, asthma, anti-asthmatic drugs, pocket depth, clinical attachment loss, periodontal risk

## Abstract

The association between asthma and periodontitis is of interest. 20 periodontitis patients with asthma (asthma group) and 20 patients without asthma (non-asthma group) were included based on inclusion and inclusion criteria. Periodontitis was classified according to 2017 periodontal classification and periodontal parameters such as tooth loss, pocket depth, clinical attachment loss, alveolar bone loss, bone reduction index, plaque index, bleeding index and periodontal risk were assessed. Effect of anti-asthmatic drugs and asthma control on periodontal parameters was also assessed. Inter-group comparison of all the continuous variables was done using independent "t" test. Comparison of categorical variables was done using Chi-square test. P value <0.05 was considered statistically significant. Results showed greater severity and higher grade of periodontitis with asthma group as well as with patients on anti-asthmatic drugs and patients with poor controlled asthma. Hence, there is an association between asthma and periodontitis.

## Background:

Asthma is a chronic multifarious disease of lower respiratory system that causes coughing and strenuous breathing [1]. Asthma results in increased morbidity among children and mortality among adults [2]. Before adolescence, the prevalence of asthma is greater among males; after adolescence, the disease's prevalence is greater among females; this contrasting effect could be due to changes in hormone levels at a young age and the use of oral contraceptives by adult patients [3]. Numerous genetic studies have emphasized the role of genetics as a causative factor of this disease [4]. As reported by various studies, genetic susceptibility to asthma ranges from 35% to 95% [5]. Despite the complex pathophysiology of asthma, evidence strongly suggests the role of inflammation in the disease's genesis and progression [1]. Therefore, it can be concluded that inflammation and infection are potential risk factors for asthma and increasingly poor prognoses [6- 7]. Current studies highlighted the presence of periodontal disease as a possible risk factor for asthma; this relationship could be due to immune and inflammatory responses or as a result of side effects caused by anti-asthma medications [8]. Poor periodontal health was also suggested to play a role [9]. However, there have been conflicting results concerning the prevalence of periodontitis in patients with asthma, including an inverse relationship showing no significant differences [10]. Some studies reported association between asthma and periodontitis [11-12]. Contradictory reports on the relationship between asthma and periodontitis are also available. Therefore, it is of interest to assess this association by determining the effects of asthma on periodontitis and periodontal risk.

## Material and Methods:

The present study included 40 periodontitis patients (10 males and 30 females) aged 21 to 55 years from Division of Periodontics, College of Dentistry, Jazan University. Before commencing the study, ethical approval was acquired from the Standing Committee for Scientific Research - Jazan University (REC-44/06/441). Written informed consent was obtained from all participants. The participants were divided into two groups: asthma group (n = 20) consisting of periodontitis patients with asthma, and non-asthma group (n = 20) consisting of periodontitis patients without asthma. Participants were excluded if they were pregnant, smokers, diagnosed as gingivitis, had any other systemic disease other than asthma, or had undergone periodontal treatment in the last 3 months.

## Asthma assessment:

Questionnaire was used to assess asthma using questions such as: "Are you diagnosed with asthma?", "Are you taking any medications for asthma?", and "How long have you had asthma?". Asthma control was sub-classified into well controlled, partly controlled, and poorly controlled based on the Global Initiative for Asthma-GINA (Global Strategy for Asthma Management and Prevention (2022 updated) [13].

## Periodontal examination and periodontitis assessment:

Periodontitis was classified according to the 2017 classification of periodontal disease [14]. Parameters such as clinical attachment loss (CAL), pocket depth, bone loss percentage, and bone loss index (calculated by bone loss percentage divided by age); bleeding score (Ainamo and Bay) [15]; and plaque score (Silness and Loe) were assessed [16]. All examinations were performed by trained dental examiners.

## Assessment of periodontal risk:

Using the number of teeth lost and the bone reduction index, the periodontal risk was assessed and categorized as low, medium, or high risk according to Lang and Tonetti [17], as follows: low risk: ≤4 missing teeth and/or bone reduction index ≤0.5; moderate risk: 5-8 missing teeth and/or bone reduction index 0.51-1.0; and high risk: ≥9 missing teeth and/or bone reduction index ≥1.1.

## Statistical analysis:

Data was analyzed using SPSS (Statistical Package for Social Sciences) 21.0 version, IBM, Chicago, USA. Data was analyzed for probability distribution using Kolmogorov-Smirnov test. Data was found to be normally distributed. Inter-group comparison of all the continuous variables was done using independent "t" test. Comparison of categorical variables was done using Chi-square test. P value <0.05 was considered statistically significant.

## Results:

In total, 40 patients (30 female and 10 male) aged between 21 to 55 years were included in the study. The mean age of the asthma group was 39.7; whereas that of the non-asthmatic group was 38.4 and the difference was non-significant with p value 0.729. The mean duration of asthma was 12.5 ± 7.4 months. 16 patients (80.0%) reported to be on medications during the same period. 13 patients (65.0%) were found to consume short-acting beta-agonists (SABA) relievers for their symptoms more than twice week. 14 patients (70.0%) reported limitations in their activities due to asthma. Based on these criteria, it was found that 13 patients (65.0%) had uncontrolled asthma, 06 (30.0%) had partially controlled asthma, and 1 (5.0%) had well-controlled asthma.

The stage of periodontitis was not found to differ significantly between the participants of the two groups (p value > 0.05). In asthma group, we found more severity of periodontitis with a greater number of patients suffering from Stage IV Periodontitis, whereas in non-asthma group, Stage II and III Periodontitis were found to be more prevalent (Table 1).

Overall, most of the patients had Grade B periodontitis. The grade of periodontitis did not yield statistically significant results between the groups (p value > 0.05). However, Grade C was found to be more common in the asthma group (Table 2).

Amount of tooth loss, bone loss percentage, plaque index, periodontal pocket depth, and bone index were greater among asthma group than non-asthma group (p value > 0.05). However, these results were non-significant. The bleeding score among asthma group was significantly greater than that among non-asthma group (p value < 0.05). Clinical attachment loss was found to be significantly more common among patients with asthma than among those without asthma (p value < 0.05) (Table 3). The results showed higher severity and grade of periodontitis with asthma group. A statistically significant high periodontal risk was found to be more common in the asthma group (Table 4). Another relevant finding was that patients on anti-asthmatic medication had more severe periodontal destruction than those who were not on medication (Table 5). When periodontal parameters were compared for the asthma control, greater periodontal destruction was found in patients having uncontrolled/partial asthma control (Table 6).

## Discussion:

The study showed that greater periodontal destruction was found among asthmatic patients than non-asthmatic patients. Greater tooth loss and bone loss percentage were found in the asthmatic patients. A significantly greater clinical attachment loss was also found in the asthmatic patients. These findings are in agreement with those of Khassawneh *et al.* [18], McDerra *et al.* [19], and Bhardwaj *et al.* [20]. However, Shulman *et al.* [21] reported no significant difference in periodontitis between asthmatic and healthy group. The asthmatic group showed greater periodontal breakdown, which occurred as a result of increased levels of immunoglobulin E (IgE) in gingival tissue, thereby resulting in a hypersensitivity reaction and decreased immunoglobulin A (IgA) levels [22,23]. Intraoral lesions may also play a role in the occurrence of asthma and other respiratory diseases due to the connectivity between the respiratory and oral cavities [24].

Deregulated immune mechanisms play a role in the pathogenesis and progression of asthma. A decrease in immune function in asthmatic patients may lead to periodontal destruction. Previous studies showed decreased IgA levels in asthmatic patients [23]. IgA plays a significant role in limiting periodontal disease. A decrease in levels of IgA levels results in periodontal destruction. The present study showed no significant plaque score differences between the asthmatic and non-asthmatic groups. This is in agreement with the results of Behal *et al.* [25]. Rivera *et al.* [26] reported no significant difference in mean plaque index among asthma patients or those using asthma drugs. Silness and Loe plaque index [16] indicates presence or absence of plaque at coronal or gingival margins but does not measure the quantity of plaque sub gingivally.

In the present study, the bleeding index was higher in asthmatics than the non-asthmatic group. These results in agreement with those of Yaghobee *et al.* [11], Laurikainen *et al.* [27], Mehta *et al.* [9] , and Khassawneh *et al.* [18]. The reasons underlying the preponderance of gingivitis in asthma are associated with alterations in immune responses and mouth breathing habits. Although the role of allergies in periodontal disease remains obscure, IgE-mediated mechanisms play a role in the destruction of periodontal tissues. Intriguingly, similar cytokines play a role in periodontal disease and the inflammatory mucous membranes of airways. Both IL-5 and IL-6 were found to be increased in asthmatic patients and those suffering from periodontitis. The inflammatory processes in periodontal disease and asthma appear to have a homogenous pathophysiology, which may, to some extent, help explain the high frequency of periodontal inflammation among asthmatics [27].

Our study found significantly higher periodontal risk among asthmatics compared to non-asthmatics, as well as increased severity of periodontal destruction in patients taking anti-asthmatic medications. Increased tooth loss, attachment loss, and bone loss were also found among patients on anti-asthmatic drugs. This could be due to mouth breathing and the frequent use of inhalational drugs among asthmatics, leading to an association between periodontal destruction and asthma [28]. Mappangara *et al.* also reported an increased risk of periodontal disease and more severe periodontitis with the use of anti-asthmatic drugs. The immunosuppressive nature of corticosteroids may have an impact on the periodontal tissue response. Another theory hypothesized to explain the increased periodontal destruction in patients taking anti-asthmatic drugs is that such drugs lead to modifications in salivary secretion, thereby negatively affecting periodontal health [28].

Our findings conflict with Grossi *et al.* [30], Arbes *et al.* [10], Friedrich *et al.* [31], Hujoel *et al.* [32], and Rivera *et al.* [26], who showed an inverse relationship between asthma and the periodontal score. The deviating inferences can be ascribed to numerous factors. Firstly, both asthma and periodontitis are multifactorial diseases. Secondly periodontal studies have been assessed differently in different studies. Gomes-Filho *et al.* [33] identified periodontitis based on clinical measures, whereas Friedrich *et al.* [31] used clinical attachment loss rather than sulcus depth to assess the severity of periodontitis. Rivera *et al.* [26] used both attachment loss and probing depth measurements to assess periodontitis. The results also differ due to disparities in the way that asthma was determined. The present study also has certain limitations such as smaller sample size, assessment of asthma was based on patient information; no lung function or challenge test were conducted to determine the severity of asthma.

## Conclusion:

Within the limitations of the study, it was found that asthma results in greater periodontal destruction than non-asthma group thereby suggesting association between asthma and periodontitis. However, further studies with larger sample size are required to determine effect of asthma on periodontal treatment.

## Ethics approval and consent to participate:

Ethical approval was acquired from the Standing Committee for Scientific Researc - Jazan University (REC-44/06/441). Informed consent was obtained from patients for participating in the study.

## Consent for publication:

Consent was obtained from the participants for publication.

## Availability of data and materials:

All data included in this published study.

## Funding:

Authors did not receive any external funding. It is a self-funded study.

## Authors' contributions

Wael I Ibraheem, Ashok Kumar Bhati, Fai Mohammed Essa Ageeli, Rehab Abdu Sufyani and Maram Ahmed Darraj, participated in conception and design of the work and data collection and data interpretation. The original draft was written by Wael I Ibraheem and Ashok Kumar Bhati. Review and editing of the draft were done by Enas Omar Ageeli, Khalid Mohammed Mobarki, Majed Yahya Alhazmi, Saiid Elshafey Mohamed Beshir. All authors made a substantial contribution to this study and/or manuscript. All authors have read and approved the final manuscript.

## Figures and Tables

**Table 1:** Distribution of study participants based on stage of periodontitis

**Stage**	**Number (%) of Participants**			
	**Asthma Group**	**Non-asthma Group**	**Total**	***p Value***
I	6 (30.0%)	4 (20.0%)	10 (25.0%)	0.595
II	3 (15.0%)	6 (30.0%)	9 (22.5%)	
III	5 (25.0%)	6 (30.0%)	11 (27.5%)	
IV	6 (30.0%)	4 (20.0%)	10 (25.0%)	
Total	20 (100.0%)	20 (100.0%)	40 (100.0%)	

**Table 2:** Distribution of study participants based on grade of periodontitis

**Grade**	**Number (%) of Participants**			
	**Asthma Group**	**Non-asthma Group**	**Total**	***p Value***
A	5 (25.0%)	1 (5.0%)	6 (15.0%)	0.06
B	9 (45.0%)	16 (80.0%)	25 (62.5%)	
C	6 (30.0%)	3 (15.0%)	9 (22.5%)	
Total	20 (100.0%)	20 (100.0%)	40 (100.0%)	
Chi-square test; p value < 0 .05 was considered statistically significant.

**Table 3:** Comparison of tooth loss, bone loss percentage, plaque index score, bleeding index, pocket depth, clinical attachment loss and Bone reduction index between asthmatics and non-asthmatics.

	**Asthma Group**		**Non-asthma Group**		***p Value***
	**Mean**	**Standard Deviation**	**Mean**	**Standard Deviation**	
Amount of tooth loss (Number)	3.7	2.193	2.1	1.943	0.13
Bone loss percentage	31.8	21.881	25.5	8.217	0.237
Plaque index	1.8	0.915	1.6	0.707	0.551
Bleeding index	61.8	23.437	46.2	19.464	0.0280 *
Pocket depth	2.69	0.489	2.71	0.527	0.877
Clinical attachment loss	3.35	0.722	2.88	0.61	0.0320 *
Bone reduction index	0.9045	0.68518	0.705	0.28543	0.343
Chi-square test; * p value < 0.05 was considered statistically significant.

**Table 4:** Distribution of study participants based on risk of periodontitis

**Periodontal Risk**	**Number (%) of Participants**			***p Value***
	**Asthma Group**	**Non-asthma Group**	**Total**	
High	8 (40.0%)	2 (10.0%)	10 (25.0%)	0.002 *
Medium	5 (25.0%)	16 (80.0%)	21 (52.5%)	
Low	7 (35.0%)	2 (10.0%)	9 (22.5%)	
Chi-square test; p value < 0 .05 was considered statistically significant.

**Table 5:** Comparison of different variables among asthmatic patients with and without medication

	**On Medication (*n* = 16)**		**Without Medication (*n* = 4)**		
	**Mean**	**Standard Deviation**	**Mean**	**Standard Deviation**	***p Value***
Amount of tooth loss	4.3	4.453	1.25	1.5	0.199
Bone loss percentage	34.8	22.711	19.6	14.325	0.222
Plaque index	1.7	0.868	1.8	1.236	0.916
Bleeding index	62.68	20.541	58.25	36.718	0.745
Periodontal pocket depth	2.67	0.529	2.75	0.331	0.792
Clinical attachment loss	3.04	0.867	2.75	0.331	0.522
Bone reduction index	0.9694	0.95772	0.645	0.51391	0.527
Independent "t" test. A p value < 0.05 was considered statistically significant.

**Table 6:** Comparison of tooth loss, bone loss percentage, clinical attachment loss and pocket depth among participants based on asthma control

			**Asthma Contol**				
	**Uncontrolled (*n* = 13)**		**Partially Controlled (*n* = 6)**		**Well Controlled (*n*=1)**		
	**Mean**	**Standard Deviation**	**Mean**	**Standard Deviation**	**Mean**	**Standard Deviation**	**p Value**
Amount of tooth loss (Number)	5.1	4.405	1.3	2.422	0	-	0.127
Bone loss percentage	37.42	24.459	24.45	6.783	12.21	-	0.31
Clinical attachment loss	3.23	0.851	2.43	0.332	3	-	0.116
Pocket depth	2.74	0.533	2.51	0.407	3	-	0.54
Independent "t" test. p value < 0.05 was considered statistically significant.
